# Supplementary material for: Effects of exchange vs. controlled diet on biochemical, body composition and functional parameters in elite female soccer players
Source: PLoS One. 2023 Nov 27;18(11):e0289114. doi: 10.1371/journal.pone.0289114 (PMC10681300; doi:10.1371/journal.pone.0289114)
Supplement: S1 Questionnaire — (DOCX) [file pone.0289114.s001.docx]

**Diet Adherence Questionnaire (AQ)**

- *Indicate your level of compliance with the diet*

|  | 1 | 2 | 3 | 4 |  |
| --- | --- | --- | --- | --- | --- |
| Have not complied with anything |  |  |  |  | Have exhaustively complied with everything |

- *Indicate which food intake has been the hardest for you to comply with.*
- Breakfast
- Mid-morning snack/lunch
- Main meal
- Afternoon snack
- Dinner
- Indicate what has been the hardest for you to eat

|  | Foods that provide carbohydrates (fruits, vegetables, legumes, cereals, pasta, and rice) | Foods that provide proteins (Meat, fish, eggs, dairy). | Everything | None of them |
| --- | --- | --- | --- | --- |
| Breakfast |  |  |  |  |
| Mid-morning snack/lunch |  |  |  |  |
| Main meal |  |  |  |  |
| Afternoon Snack |  |  |  |  |
| Dinner |  |  |  |  |

- Indicate which meal you have usually skipped (you can select several answers).
- Berakfast
- Mid-morning snack/lunch
- Main meal
- Afternoon snack
- Dinner
- None
- Indicate if any of the meals seem excessive to you (you can select several answers)

- Berakfast
- Mid-morning snack/lunch
- Main meal
- Afternoon snack
- Dinner
- Indicate if any of the meals seem insufficient to you (you can select several answers)
- Berakfast
- Mid-morning snack/lunch
- Main meal
- Afternoon snack
- Dinner
- Indicate the degree of difficulty that following the diet has entailed for you

|  | 1 | 2 | 3 | 4 | 5 | 6 | 7 | 8 | 9 | 10 |  |
| --- | --- | --- | --- | --- | --- | --- | --- | --- | --- | --- | --- |
| It has not been difficult for me |  |  |  |  |  |  |  |  |  |  | **It has been extremely difficult for me** |

- What has been your biggest challenge (you can select several answers).
- Cooking
- Calculate amounts
- Vary menus.
- Completing al lof the meals
- I have no difficulties at all

**Diet Adherence Questionnaire (AQ)**

- *Indicate your level of compliance with the diet*

|  | 1 | 2 | 3 | 4 |  |
| --- | --- | --- | --- | --- | --- |
| Have not complied with anything |  |  |  |  | Have exhaustively complied with everything |

- *Indicate which food intake has been the hardest for you to comply with.*
- Breakfast
- Mid-morning snack/lunch
- Main meal
- Afternoon snack
- Dinner
- Indicate what has been the hardest for you to eat

|  | Carbohydrate equivalents | Protein equivalents | Everything | None of them |
| --- | --- | --- | --- | --- |
| Breakfast |  |  |  |  |
| Mid-morning snack/lunch |  |  |  |  |
| Main meal |  |  |  |  |
| Afternoon Snack |  |  |  |  |
| Dinner |  |  |  |  |

- Indicate which meal you have usually skipped (you can select several answers).
- Berakfast
- Mid-morning snack/lunch
- Main meal
- Afternoon snack
- Dinner
- None
- Indicate if any of the meals seem excessive to you (you can select several answers)

- Berakfast
- Mid-morning snack/lunch
- Main meal
- Afternoon snack
- Dinner
- Indicate if any of the meals seem insufficient to you (you can select several answers)
- Berakfast
- Mid-morning snack/lunch
- Main meal
- Afternoon snack
- Dinner
- Indicate the degree of difficulty that following the diet has entailed for you

|  | 1 | 2 | 3 | 4 | 5 | 6 | 7 | 8 | 9 | 10 |  |
| --- | --- | --- | --- | --- | --- | --- | --- | --- | --- | --- | --- |
| It has not been difficult for me |  |  |  |  |  |  |  |  |  |  | **It has been extremely difficult for me** |

- What has been your biggest challenge (you can select several answers).
- Cooking
- Calculate amounts
- Vary menus.
- Completing al lof the meals
- I have no difficulties at all
