# Supplementary material for: Effects of exchange vs. controlled diet on biochemical, body composition and functional parameters in elite female soccer players
Source: PLoS One. 2023 Nov 27;18(11):e0289114. doi: 10.1371/journal.pone.0289114 (PMC10681300; doi:10.1371/journal.pone.0289114)
Supplement: S2 Questionnaire — (DOCX) [file pone.0289114.s002.docx]

**Cuestionario de adherencia a la dieta (CG)/** **Diet Adherence Questionnaire (AQ)**

- Señala cuál ha sido tu grado de cumplimiento de la dieta/*Indicate your level of compliance with the diet*

|  | 1 | 2 | 3 | 4 |  |
| --- | --- | --- | --- | --- | --- |
| No he cumplido nada |  |  |  |  | He cumplido todo exhaustivamente |

|  | 1 | 2 | 3 | 4 |  |
| --- | --- | --- | --- | --- | --- |
| Have not complied with anything |  |  |  |  | Have exhaustively complied with everything |

- Señala qué ingesta te ha costado más cumplir/*Indicate which food intake has been the hardest for you to comply with.*
- Desayuno/Breakfast
- Almuerzo/Mid-morning snack/lunch
- Comida /Main meal
- Merienda /Afternoon snack
- Cena/Dinner
- Señala qué te ha costado más comer/Indicate what has been the hardest for you to eat

|  | alimentos que aportan hidratos (frutas, verduras, legumbres, hortalizas, cereales, pasta y arroz) | Alimentos que aportan proteína (Carne, pescado, huevos, lácteos) | Todo en general | no me ha costado ninguno |
| --- | --- | --- | --- | --- |
| Desayuno |  |  |  |  |
| Almuerzo |  |  |  |  |
| Comida |  |  |  |  |
| Merienda |  |  |  |  |
| Cena |  |  |  |  |

|  | Foods that provide carbohydrates (fruits, vegetables, legumes, cereals, pasta, and rice) | Foods that provide proteins (Meat, fish, eggs, dairy). | Everything | None of them |
| --- | --- | --- | --- | --- |
| Breakfast |  |  |  |  |
| Mid-morning snack/lunch |  |  |  |  |
| Main meal |  |  |  |  |
| Afternoon Snack |  |  |  |  |
| Dinner |  |  |  |  |

- Señala qué ingesta te has saltado habitualmente (puedes seleccionar varias respuestas)/ Indicate which meal you have usually skipped (you can select several answers).
- Desayuno/Berakfast
- Almuerzo/Mid-morning snack/lunch
- Comida/ Main meal
- Merienda / Afternoon snack
- Cena /Dinner
- Ninguna/ None
- Señala si alguna de las ingestas te parece excesiva (puedes señalar varias respuestas)/Indicate if any of the meals seem excessive to you (you can select several answers)

- Desayuno/Berakfast
- Almuerzo/Mid-morning snack/lunch
- Comida/ Main meal
- Merienda / Afternoon snack
- Cena /Dinner
- Señala si alguna de las ingestas te parece escasa (puedes señalar varias)/ Indicate if any of the meals seem insufficient to you (you can select several answers)
- Desayuno/Berakfast
- Almuerzo/Mid-morning snack/lunch
- Comida/ Main meal
- Merienda / Afternoon snack
- Cena /Dinner
- Señala el grado de dificultad que te ha supuesto seguir la dieta/Indicate the degree of difficulty that following the diet has entailed for you

|  | 1 | 2 | 3 | 4 | 5 | 6 | 7 | 8 | 9 | 10 |  |
| --- | --- | --- | --- | --- | --- | --- | --- | --- | --- | --- | --- |
| No me ha resultado difícil |  |  |  |  |  |  |  |  |  |  | **Me ha resultado extremadamente difícil** |

|  | 1 | 2 | 3 | 4 | 5 | 6 | 7 | 8 | 9 | 10 |  |
| --- | --- | --- | --- | --- | --- | --- | --- | --- | --- | --- | --- |
| It has not been difficult for me |  |  |  |  |  |  |  |  |  |  | **It has been extremely difficult for me** |

- Cuál ha sido tu mayor dificultad (puedes señalar varias respuestas)/What has been your biggest challenge (you can select several answers).
- Cocinar/Cooking
- calcular cantidades/Calculate amounts
- variar menús/Vary menus.
- completar todas las ingestas/Completing al lof the meals
- no he tenido dificultades en nada/ I have no difficulties at all

**Cuestionario de adherencia a la dieta (EG)/** **Diet Adherence Questionnaire (AQ)**

- Señala cuál ha sido tu grado de cumplimiento de la dieta/*Indicate your level of compliance with the diet*

|  | 1 | 2 | 3 | 4 |  |
| --- | --- | --- | --- | --- | --- |
| No he cumplido nada |  |  |  |  | He cumplido todo exhaustivamente |

|  | 1 | 2 | 3 | 4 |  |
| --- | --- | --- | --- | --- | --- |
| Have not complied with anything |  |  |  |  | Have exhaustively complied with everything |

- Señala qué ingesta te ha costado más cumplir/*Indicate which food intake has been the hardest for you to comply with.*
- Desayuno/Breakfast
- Almuerzo/Mid-morning snack/lunch
- Comida /Main meal
- Merienda /Afternoon snack
- Cena/Dinner
- Señala qué te ha costado más comer/Indicate what has been the hardest for you to eat

|  | Bloques de HCO | Bloques de Prot | Todo en general | no me ha costado ninguno |
| --- | --- | --- | --- | --- |
| Desayuno |  |  |  |  |
| almuerzo |  |  |  |  |
| comida |  |  |  |  |
| merienda |  |  |  |  |
| cena |  |  |  |  |

|  | Carbohydrate equivalents | Protein equivalents | Everything | None of them |
| --- | --- | --- | --- | --- |
| Breakfast |  |  |  |  |
| Mid-morning snack/lunch |  |  |  |  |
| Main meal |  |  |  |  |
| Afternoon Snack |  |  |  |  |
| Dinner |  |  |  |  |

- Señala qué ingesta te has saltado habitualmente (puedes seleccionar varias respuestas)/ Indicate which meal you have usually skipped (you can select several answers).
- Desayuno/Berakfast
- Almuerzo/Mid-morning snack/lunch
- Comida/ Main meal
- Merienda / Afternoon snack
- Cena /Dinner
- Ninguna/ None
- Señala si alguna de las ingestas te parece excesiva (puedes señalar varias respuestas)/Indicate if any of the meals seem excessive to you (you can select several answers)

- Desayuno/Berakfast
- Almuerzo/Mid-morning snack/lunch
- Comida/ Main meal
- Merienda / Afternoon snack
- Cena /Dinner
- Señala si alguna de las ingestas te parece escasa (puedes señalar varias)/ Indicate if any of the meals seem insufficient to you (you can select several answers)
- Desayuno/Berakfast
- Almuerzo/Mid-morning snack/lunch
- Comida/ Main meal
- Merienda / Afternoon snack
- Cena /Dinner
- Señala el grado de dificultad que te ha supuesto seguir la dieta/Indicate the degree of difficulty that following the diet has entailed for you

|  | 1 | 2 | 3 | 4 | 5 | 6 | 7 | 8 | 9 | 10 |  |
| --- | --- | --- | --- | --- | --- | --- | --- | --- | --- | --- | --- |
| No me ha resultado difícil |  |  |  |  |  |  |  |  |  |  | **Me ha resultado extremadamente difícil** |
|  |  |  |  |  |  |  |  |  |  |  |  |

|  | 1 | 2 | 3 | 4 | 5 | 6 | 7 | 8 | 9 | 10 |  |
| --- | --- | --- | --- | --- | --- | --- | --- | --- | --- | --- | --- |
| It has not been difficult for me |  |  |  |  |  |  |  |  |  |  | **It has been extremely difficult for me** |

- Cuál ha sido tu mayor dificultad (puedes señalar varias respuestas)/What has been your biggest challenge (you can select several answers).
- Cocinar/Cooking
- Calcular cantidades/Calculate amounts
- Variar menús/Vary menus.
- Completar todas las ingestas/Completing al lof the meals
- No he tenido dificultades en nada/ I have no difficulties at all
