# Supplementary material for: Effects of exchange vs. controlled diet on biochemical, body composition and functional parameters in elite female soccer players
Source: PLoS One. 2023 Nov 27;18(11):e0289114. doi: 10.1371/journal.pone.0289114 (PMC10681300; doi:10.1371/journal.pone.0289114)
Supplement: Attachment — Submitted filename: response to reviewers.docx [file pone.0289114.s005.docx]

- Manuscript PONE-D-22-26202: *Effects of exchange vs. controlled diet on biochemical, body composition and functional parameters in elite female soccer players*

Response to reviewers (ACADEMIC EDITOR). Please find our anwers, point by point:

**Point 1 (Sample size)**

*Accurate calculations and their screens in terms of the correctness of the determination of the minimum sample size should be provided. The indicated sample size may refer to the size within one given group. In this case, numbers of 9 or 10 in the group would not be sufficient.*

**Response: We appreciate this comment. We have made the pertinent modifications to clarify this point in the second paragraph of the Statistical Analysis section. In addition, it should be noted that the sample is highly representative of the study population, as it includes 19 players of a total of 327 federated female soccer players within the highest Spanish soccer level.**

**Point 2 (Accuracy of data)**

*Accuracy of data should be reported in accordance with the accuracy of the method.*

**Response: We have added the intraclass correlation coefficient (ICC) values in order to show the accuracy of the data. It can be found in the METHODS Section, both for anthropometric measurements and physical performance. Moreover, in the Statistical analysis section we have indicated that “the reliability of measurements was calculated using the ICC”.**

**Point 3 (decimal places)**

*Thus, the energy supply in kcal should not have any decimal places at all. Similarly, for 3-digit nutrient intake values are overestimated - this should be corrected.*

**Response: According to you comment, we have checked and corrected all the manuscript and tables.**

**Point 4 (Correct entries and editing)**

*Correct the correct entries and editing - e.g. superscripts in some units (g·kg^-1^), data spacing etc.*

**Response: According to you comment, we have checked and corrected all the manuscript and tables.**

**Point 5 (Flowchart)**

*Including a flowchart of the study design and procedure will be beneficial to consider.*

**Response: As you suggested, we have added a flowchart of the study in order to clarify the information given. It has been included as Figure 1 in the METHODS section.**
