# Supplementary material for: Effects of exchange vs. controlled diet on biochemical, body composition and functional parameters in elite female soccer players
Source: PLoS One. 2023 Nov 27;18(11):e0289114. doi: 10.1371/journal.pone.0289114 (PMC10681300; doi:10.1371/journal.pone.0289114)
Supplement: Attachment — Submitted filename: Response to reviewers.docx [file pone.0289114.s006.docx]

**Manuscript PONE-D-22-26202R1 (Review 1): answers to the referees.**

**Answers to reviewer 1**

**Please find our answers point by point. The changes are highlighted in the manuscript.**

1. **The recommendations used in table 2 should be precisely described in methods, especially the estimation of calory needs.**
2. **Line 181: It is unclear how the authors chose the level of nutritional needs. The CHO differs from the cited recommendations and table 2, and it is unclear why they chose such levels of PROT and LIP. Also, the kcal level in table 1 is too low to satisfy the energy needs of athletes in this study.**

As you suggested, we have made some changes in the *Methods* section in order to make it clearer (Lines:130-138). Additionally, we have added some information in the table caption (table 2).

1. **In methods: the information about controlled and exchange diet should be precisely describe: precise characteristics and differences.**

Following your advice, we have explained with more detail both dietetic interventions. (Lines: 142-154). (Methods section/Design and procedures)

2. **I propose not to use abbreviations in the titles of tables and sections.**

We have made some changes along the *manuscript to solve this problem*.

3. **Table 2. The authors should describe that the values presented in table 2 are calculated according to a different number of days: the weekdays –5 days, and pre-competition and competition days – one day. The way of calculation should be described in the methods and also in the information under the table.**

In order to clarify this point, some changes have been made in the table (title and table caption) and in the text (Line: 159/ Methods section/Measures/ Dietary intake, nutritional knowledge and adherence questionnaires).

4. **Table 2. Under the table, the citations for the recommendations used should be included.**

The references have been included under the table.

**Answers to reviewer 2**

**Please find our answers point by point**

**Reviewer #2: Minor revisions
1. Line 23: Add ‘players’ or ‘athletes’ after ‘elite female soccer’.
2. Line 33: Add comma before the word ‘while’.**

**5.Line 53: The word ‘achieved’ seems to be out of the context and could be removed.**

**6. Line 55: Change ‘needs’ into ‘need’.**

**9. Line 90: Use italics for ‘a priori’.
10. Unify using/not using spaces between special signs such as ‘+/=/±/’ within the whole manuscript.
11. Line 108: The ‘,’ is not necessary.
12. Line 198: Correct the writing of ‘guidelines’.**

**14. Table 2: Please change the language into English in the recommendations for fibre intake (now it states 22 - 25 g/día)**

We have followed all the instructions given for the reviewer. Some of them were trivial mistakes that have been corrected. Thank you very much.

**3.Line 36: The expression ‘deficiency of haemoglobin’ seems to debatable. Consider using the expression ’participants presented decreased concentration of haemoglobin’ or ‘concentration of haemoglobin was below the reference level’.**

Thank you very much for your helpful advice. It sounds much better that way.

**4.Please explain all the abbreviations when they are first mentioned in the text. Keep using the same abbreviation for the corresponding expression throughout the whole manuscript (including tables, figures and text).**

The entire manuscript has been reviewed to correct any mistakes.

**7. Line 80 – 83: No reference number is provided!**

The reference number is now provided. (Line: 81)

**8. Line 83: The sentence ‘There are different nutritional intervention strategies to use with population’ is not clear.**

The sentence has been rewritten to make it clearer. (Line: 84).

**13. Using ‘LIP’ abbreviation for dietary fat is debatable. I would decline from using the abbreviation.**

Following your suggestion, we have replaced all the “LIP” abbreviation for the word “fat” which sounds better.

**15. The recording of Reference list must to unified according to the guide lines of the journal.**

The reference list has been unified.

**Reviewer #2: Major revisions**

**1.The manuscript must undergo extensive English proof reading by native speaker.**

The manuscript has been reviewed, and many changes have been made to improve the language. However, not all the changes have been highlighted at this point to make the text easier to read.

**2.The writing of units needs to be unified within the whole manuscript!**

**6.Two different abbreviations are used to ‘exchange diet’, namely ‘EG’ and ‘ED’ – it must be unified!**

**8. Unify the number of decimal places for the same variables within the whole manuscript. The examples of mistakes with this respect can be found in lines 102 – 105, and other parts of the manuscript as well.**

The entire manuscript has been reviewed to correct any mistakes.

**3.Line 59 – 60: Please consider referring to the following paper and recommendations: 10.1186/s12970-018-0242-y**

**5. Line 66 – 67: Please consider referring to the following paper and recommendations: 10.1186/s12970-018-0242-y**

Thank you very much for your advice. We have added the reference mentioned.

**4. Line 63 – 65: The given intake of PROT in females soccer players ‘1.7–2 g·kg-1 per day’ seems to be within the stated in the former part of manuscript (Line 61) recommendation range (i.e. 1.2 and 2.5 g·kg-1 per day), and not higher than recommendations (as Authors stated). The information requires verification and explanation**.

Thank you very much for the appreciation. There was an error in the text that has been corrected.

**7. Figure 1 is not clear at all! It is not known which allocation refers to ‘CD’, and which to ‘ED/EG’. It must be improved.**

Thank you very much for your  assessment. We have added the corresponding group allocation in the participan flow diagram to improve it.

**9. Please provide the questions that were included in the Diet Adherence Questionnaire (AQ). They are not provided at any part of the manuscript, and the questionnaire is new and not validated in the previous literature. Authors may provide the AQ as a supplementary material**.

The document has been attached as a supplementary file.

**10. Line 119: Please provide the reference which describe the implemented methodology of dietary recording.**

**11. Line 124: Please provide the reference for EFSA guidelines that are mentioned in the text.**

**13. Provide the reference for ‘the International Society for the Advancement in Kinanthropometry’ (Line 140).**

**16. Provide the reference for the methodology of physical performance tests (i.e., running speed and CMJA).**

These references have been included. (Reference list, numbers 19, 21, 24, 25 and 26, respectively).

**12. Line 130: There is a mistake in percentage classification of NK, to specify the mistake refers to the value**

Sorry, it was a trivial mistake that has been corrected.

**14. Line 161: How was euhydration state verified?**

The euhydration status of the players prior to the blood analyses was not specifically verified. However, those players strictly followed the protocols established by the laboratory resposinble of the test, which teoretically guarantee proper hydration status. In order to avoid misunderstunding, the word “euhydrated” has been omitted. (Line 202)

**15. Consider moving the paragraph ‘Design and Procedures’ right after the paragraph ‘Participants’**.

The change suggested has been made.

**17. Line 185: What does the abbreviation ‘CG’ stands for?**

All the abreviations have been revised in order to avoid misunderstandings.

**18. Statistical analysis – did all the variables actually present the normal distribution of the data? What was the procedure for non-normal distributed data?**

All the variables presented a normal distribution of the data, therefore, we did not use any procedure for non normal distributed data. We have made some changes in the text in order to clarify this point (Line 211).

**19. The references for the recommendations provided in the Table 2 must be given.**

The references have been included under the table.

**20. Please correct the writing of the units in the Table 2**.

The entire manuscript has been reviewed to correct the units.

**21. Table 2: Please provide the exact equations that were used for estimating energy requirements!**

We have made some changes in the Methods section in order to make it clearer (Lines:130-138). Additionally, we have added some information in the table caption.

**22. Correct and unify decimal places (within the same variable) in the Table 2.**

The entire manuscript has been reviewed to unify decimal places.

**23. Table 3: The literature for each of reference range for each particular blood indices need to be provided.**

The reference ranges used for the parameters of the blood análisis are established by the laboratory responsable for peforming them. That is the reason why we have not included any reference to them, as we understand that they are sufficiently validated intervals.
