# Supplementary material for: Effects of exchange vs. controlled diet on biochemical, body composition and functional parameters in elite female soccer players
Source: PLoS One. 2023 Nov 27;18(11):e0289114. doi: 10.1371/journal.pone.0289114 (PMC10681300; doi:10.1371/journal.pone.0289114)
Supplement: Attachment — Submitted filename: Response to Reviewers.docx [file pone.0289114.s007.docx]

**Manuscript PONE-D-22-26202R2(Revision 2): answers to the referees.**

**Answers to reviewer 2**

**Please find our answers point by point. The changes are highlighted in the manuscript.**

**MINOR REVISIONS**

1. **Please unify writing of heamoglobin/hemoglobin within the whole manuscript (an example of various writing can be found in the lines 36 – 37; please verify the writing of the related words, e.g. hematopoietic).**
2. **Line 39: I suggest replacing “parameters” with “indices”. Same in the line 122.**
3. **Do not use the space between the value and “%” sign.**
4. **Line 54: I suggest adding “physical” before “performance”.**

We have followed all the instructions provided by the reviewer. Some of them were trivial mistakes that have been corrected. Thank you very much for your guidance.

5. **Line 60: There is no position no. 8 in the reference list.**

The reference list was reviewed, and a new reference (no.8) was added, as it was unintentionally skipped.

6. **I suggest using 5 – 7 gCHO·kgBM-1∙day-1 instead of 5 – 7 g·kg-1 per day (I suggest analogous changes within the whole manuscript with regard to “similar” units).**
7. **Line 64: You can use “PROT” for protein (please revise the whole manuscript with this respect)**

The entire manuscript has been reviewed and corrections have been made to address your suggestions.

8. **Line 67-68: I can’t agree with the statement.**

In the statement made in lines 67-68, we simply refer to what has been indicated in previous studies, which have observed that the fat intake in female soccer players falls within the recommended range of 20-35%. However, within that intake, the consumption of saturated fats generally exceeds the recommended limit of 10%. This is also observed in the present study, although it is not mentioned explicty yet, as it will be included in the *Results* and *Discussion* sections later on.

9. **Line 115: Figure 1 shows the flow of participants in the study – the sentence is redundant.**
10. **Line 116. Correct the writing of the title of the Figure 1.**

Following your recomendations, the sentence has been omitted and the title corrected.

11. **Line 130: Correct the writing of “kg”.**

**13.Table 1. Replace “,” with “.”.**

**16. Line 387: “dietetic” must be replace with “dietary”.**

All of these minor corrections have already been made.

12. **Authors need to indicate in the text of the manuscript that the “Adherence questionnaire” is provided as an attachment. It should be provided also in English language version.**

As you suggested, it is indicated in lines 176-177 and the adherence questionnaire is now provided in English. Previously, it was provided in both Spanish and English.

14. **Lines 232-237: I suggest providing the full names of mineral (at least when first mention in the text).**

The entire manuscript has been reviewed and corrections have been made to follow your suggestion.

15. **The reference list is still in mess. It must be improved.**

The entire reference list has been thoroughly reviewed and several mistakes have been corrected.

17. **I recommend providing results of vertical jump in cm.**

As you suggested, the results are now provided in cm (Figure 2).

**MAJOR REVISIONS**

**1. Line 105: How was the randomization process performed?**

As it is an important question, we have added an explanation in the text (lines 107-108). A simple randomization method was employed by generating a table of random numbers without repetition. Participants with even numbers were assigned to the CG, while participants with odd numbers were assigned to the EG.

**2. Line 124: Why was dietary intake recorded only PRE? It seems to be a serious methodological concern.**

While it could be considered a serious methodological concern, the main objective of the study was not to evaluate changes in dietary intake post-intervention. Instead, the focus was on assessing the nutritional energy intake pre-intervention for comparison with general recommendations and for the purpose of implementing an appropriate nutritional intervention. However, it is important to acknowledge that not evaluating post-intervention dietary intake may limit the assessment of the intervention’s effectiveness in terms of dietary changes.

**3. Line 130 – 131: Energy intake during dietary intervention was calculated based of an average body mass. It is a serious impropriety. It must have been adjusted to each participant individual body mass. Same should be done with regard to CHO, PROT and FAT.**

It is important to address the concern raised by the reviewer regarding individual variations in body mass. However, it is crucial to consider the context of the present study, which was an initial group intervention aimed at assessing suitable intervention methodologies based on adherence and nutritional knowledge questionnaire results. Given that most players had weights close to the average, using the average weight to meet the nutritional requirements of all players was initially considered appropriate. Nevertheless, upon reflection, we acknowledge that adjusting energy intake to each participant’s individual body mass would have provided a more precise and tailored approach to meet their specific needs. We apologize for the oversight and recognize the importance of individualizing nutritional interventions in future studies. In light of the results obtained in the present study, we intend to implement interventions based on each player’s individual objectives, periodized according to the training plan and the stage of the season. Additionally, we plan to incorporate periodization based on each player’s menstrual cycle.

Thank you for bringing this concern to our attention, and we appreciate the opportunity to improve our methodology for future research.

**4. Lines 137:138. I do not agree with the statement “A sudden increase in CHO intake would have been difficult to adhere to”. The assumptions of implemented dietary intervention should take into account actual needs for CHO and the intake of CHO during intervention should be in line with current scientific-based recommendations. This fact must be discussed in the Discussion paragraph as a serious limitation of the study.**

Thank you for your feedback. As you suggested, we have included a paragraph (lines 397-405) in the limitations section of the study (Discussion) to address the issue of carbohydrate intake during the intervention, which was slightly lower than the current minimum recommendation. This decision was made to ensure gradual changes in carbohydrate intake during the intervention, taking into consideration the high prevalence of eating disorders and the focus on weight control among female athletes. By avoiding a sudden increase in intake, we aimed to create a more manageable and sustainable approach for the participants. By acknowledging this as a limitation, we emphasize the importance of future research considering the participant’s actual needs, aligning their carbohydrate intake with current scientific-based recommendations during intervention.

**5. Line 205-206: “The evaluation was based on the reference range (table 3) indicated by the 206 biochemical analysis laboratory” – still, the literature references for the ranges must be provided**.

Thank you for your additional comments regarding the reference ranges used in the study. The laboratory used for the analisis was Synlab, a clinical laboratory accredited by national accreditation bodies such as DAkkS in Germany, SAS in Switzerland, and ENAC in Spain. These bodies evaluate and accredit laboratories to ensure their technical competence and compliance with relevant international and national standards. You can find more information about Synlab and its accreditations on their oficial website: <https://synlab.es> . Unfortunately, specific literature references for the reference ranges used in the clinical laboratory were not included, something that is commeon in scientific articles of this kind, as the information provided is based on the laboratory’s internal standards and protocols in accordance with its accreditation.

**6. Statistical analysis: I would kindly ask the Authors to provide the raw data of study results for the for inspection of reviewers.**

In order to facilitate the comprehension of Figure 2, the raw data have been attached as a supplementary file, entitled FIGURE 2 DATABASE.

**7. Table 2: It is not necessary to provide two decimal places with regard to the intake of CHO, PROT, fat, fatty acids when expressed as g∙day-1; fiber, calcium, phosphorus, magnesium, iron, vitamin C.**

Following your suggestion, we have reviewed the entire table 2 and made the necessary changes.

**8. Lines 300-302: “Regarding biochemical values, a well-established controlled diet, including Fe and vit C consumption (34), has shown to increase haemoglobin concentration but no other parameters” - there is no justification for such a statement, while the intakes of Fe and Vit C were not monitored, neither during the intervention, nor POST-intervention. We don not know the exact reason for increase in haemoglobin concentration.**

This statement has been omitted in this section, as it should exclusively focus on presenting results without interpretations. However, we have added a possible explanation in the discussion section (lines 372-378) to address this aspect.

**9. Lines 310-325: Author underline the importance of CHO in the nutrition of female soccer players and the assumptions of the implemented dietary interventions (EG and CD) did not ensure provision of CHO according to recommendations.**

As stated previously in point 4 of the major revisions, we have acknowledged this as a limitaion os the study. A paragraph has been added in the duscussion section to adress this subject.

**10. Sample size calculation: Authors stated “ The estimated sample size was 17 participants (actual power = 0.802), but considering possible dropouts, we enrolled 19 participants in this study” (lines 226-227). While in the Figure 1 it is said that 21 participants were enrolled, 20 were randomized, and there was 1 drop-out. It is thus inconsistent.**

Following your comment, we have carefully reviewed both the text and Figure 1 and identified an error in the graphic. We have made the necessary changes to correct it. Additionally, we have made clarifications in the text (lines 220-234) to provide a clearer explanation of the sample size calculation.
