# Supplementary material for: Effects of exchange vs. controlled diet on biochemical, body composition and functional parameters in elite female soccer players
Source: PLoS One. 2023 Nov 27;18(11):e0289114. doi: 10.1371/journal.pone.0289114 (PMC10681300; doi:10.1371/journal.pone.0289114)
Supplement: Attachment — Submitted filename: Response to reviewers.docx [file pone.0289114.s008.docx]

- Manuscript PONE-D-22-26202: *Effects of exchange vs. controlled diet on biochemical, body composition and functional parameters in elite female soccer players* (Revision 3): answers to the referees.

**Answer to reviewer 2**

**COMMENTS**

**Although Authors addressed carefully most of my previous revisions, one of the main major revision is still not resolved. Namely, Authors provided the data set solely to support Figure 2. Remaining data sets are not provided. Thus, I would kindly ask to provide data supporting all the remaining tables and figures for review process only.**

**We sincerely apologize for our previous response, because we misunderstood your requirements. We thought you were only referring to raw data for Figure 2. As per your suggestion, we have now attached a supplementary file titled “GLOBAL DATABASE”.**
